# Comparing Methods of Assessing Dog Rabies Vaccination Coverage in Rural and Urban Communities in Tanzania

**DOI:** 10.3389/fvets.2017.00033

**Published:** 2017-03-14

**Authors:** Maganga Sambo, Paul C. D. Johnson, Karen Hotopp, Joel Changalucha, Sarah Cleaveland, Rudovick Kazwala, Tiziana Lembo, Ahmed Lugelo, Kennedy Lushasi, Mathew Maziku, Eberhard Mbunda, Zacharia Mtema, Lwitiko Sikana, Sunny E. Townsend, Katie Hampson

**Affiliations:** ^1^Environmental Health and Ecological Sciences Thematic Group, Ifakara Health Institute, Ifakara, Tanzania; ^2^Boyd Orr Centre for Population and Ecosystem Health, Institute of Biodiversity, Animal Health and Comparative Medicine, College of Medical, Veterinary and Life Sciences, University of Glasgow, Glasgow, UK; ^3^College of Veterinary and Medical Sciences, Sokoine University of Agriculture, Morogoro, Tanzania; ^4^Ministry of Agriculture, Livestock and Fisheries Development, Dar Es Salaam, Tanzania

**Keywords:** rabies, rabies control, accuracy, dog vaccination, rabies elimination, dog rabies

## Abstract

Rabies can be eliminated by achieving comprehensive coverage of 70% of domestic dogs during annual mass vaccination campaigns. Estimates of vaccination coverage are, therefore, required to evaluate and manage mass dog vaccination programs; however, there is no specific guidance for the most accurate and efficient methods for estimating coverage in different settings. Here, we compare post-vaccination transects, school-based surveys, and household surveys across 28 districts in southeast Tanzania and Pemba island covering rural, urban, coastal and inland settings, and a range of different livelihoods and religious backgrounds. These approaches were explored in detail in a single district in northwest Tanzania (Serengeti), where their performance was compared with a complete dog population census that also recorded dog vaccination status. Post-vaccination transects involved counting marked (vaccinated) and unmarked (unvaccinated) dogs immediately after campaigns in 2,155 villages (24,721 dogs counted). School-based surveys were administered to 8,587 primary school pupils each representing a unique household, in 119 randomly selected schools approximately 2 months after campaigns. Household surveys were conducted in 160 randomly selected villages (4,488 households) in July/August 2011. Costs to implement these coverage assessments were $12.01, $66.12, and $155.70 per village for post-vaccination transects, school-based, and household surveys, respectively. Simulations were performed to assess the effect of sampling on the precision of coverage estimation. The sampling effort required to obtain reasonably precise estimates of coverage from household surveys is generally very high and probably prohibitively expensive for routine monitoring across large areas, particularly in communities with high human to dog ratios. School-based surveys partially overcame sampling constraints, however, were also costly to obtain reasonably precise estimates of coverage. Post-vaccination transects provided precise and timely estimates of community-level coverage that could be used to troubleshoot the performance of campaigns across large areas. However, transects typically overestimated coverage by around 10%, which therefore needs consideration when evaluating the impacts of campaigns. We discuss the advantages and disadvantages of these different methods and make recommendations for how vaccination campaigns can be better monitored and managed at different stages of rabies control and elimination programs.

## Introduction

Rabies is a fatal viral disease transmitted to humans by animal bites, usually from domestic dogs. Although under control in most industrialized countries, rabies continues to kill an estimated 59,000 people each year in low- and middle-income countries (LMICs) (1). Reliable estimates of the proportion of dogs vaccinated against rabies are crucial to determine the performance of vaccination programs and their impact on disease transmission. Empirical and theoretical evidence shows that mass dog vaccination campaigns that reach at least 70% of the dog population can control rabies (2, 3). While achieving this coverage in all communities can lead to elimination, even small gaps in coverage can delay the time to elimination (4). As progress is made toward reaching global targets of zero human rabies deaths from dog-mediated rabies through the implementation of mass dog vaccinations (5), there is a clear need to identify reliable, cost-effective, and feasible approaches that can be used, at scale, to assess community-level vaccination coverage.

Limited population data on owned and free-roaming dogs in most LMICs make estimation of vaccination coverage challenging. Several methods have been used to estimate coverage including (i) the use of pre-campaign estimates of dog population size through human to dog ratios (HDRs) as the denominator, and the number of dogs vaccinated during the campaign as the numerator (6); (ii) post-vaccination household surveys to estimate the proportion of vaccinated dogs (7–11); and (iii) post-vaccination transects to estimate the proportion of marked (vaccinated) dogs (4, 12–14). However, these methods all have limitations.

If dog populations are estimated from data on HDRs, inaccuracies in estimates of the human population will invariably affect the accuracy of dog population estimates. This may occur, for example, through errors in extrapolating current human population sizes from census data (for example, using average population growth rates) or from administrative/boundary changes that affect village demarcations across different time periods. Furthermore, published data on HDRs usually reflect a sample from surveys across several communities (15), and even a small degree of variation in HDRs can have a major effect on dog population estimates at the community level.

Household surveys are restricted to capturing estimates of vaccination coverage in owned dog populations and are relatively intensive to complete. Moreover, there is known to be wide variability in patterns of dog ownership within communities—for example, in Tanzania, a much smaller proportion of Muslim and urban households own dogs in comparison with rural, livestock-keeping communities (15). This variability and the highly skewed pattern of dog ownership in some communities make household surveys prone to selection and measurement biases (16). Additional uncertainty from household surveys arises in relation to validation of dog vaccination status. In Tunisia, for example, about 14% of dog owners who claimed their dogs were vaccinated were unable to provide certificates (17).

Post-vaccination transects are limited to observations of free-roaming dogs and will, therefore, be biased toward dogs that are more likely to be observed from transects. For example, young puppies are likely to be less visible and are known to represent an age group that typically has a low vaccination coverage (9, 18, 19), thus resulting in the potential for overestimating coverage. In a recent study from Tanzania, post-vaccination transects were shown to overestimate coverage by approximately 7% in comparison with household surveys, although it was unclear in this study which of the approaches was most accurate (19).

Here, we present a detailed assessment of three methods to estimate dog vaccination coverage across settings in Tanzania. We use a complete household census as reference data for a simulation experiment to determine the impacts of sampling on the precision of coverage estimates. Specifically, we aim to answer the following questions: (i) What are the resources (personnel, time, and money) required to implement these methods? (ii) Which methods provide the most precise estimates of coverage? and finally (iii) Which approaches, therefore, generate acceptable coverage estimates to provide operational guidance to improve the performance of current or future campaigns?

## Materials and Methods

### Study Sites

**Figure 1:**
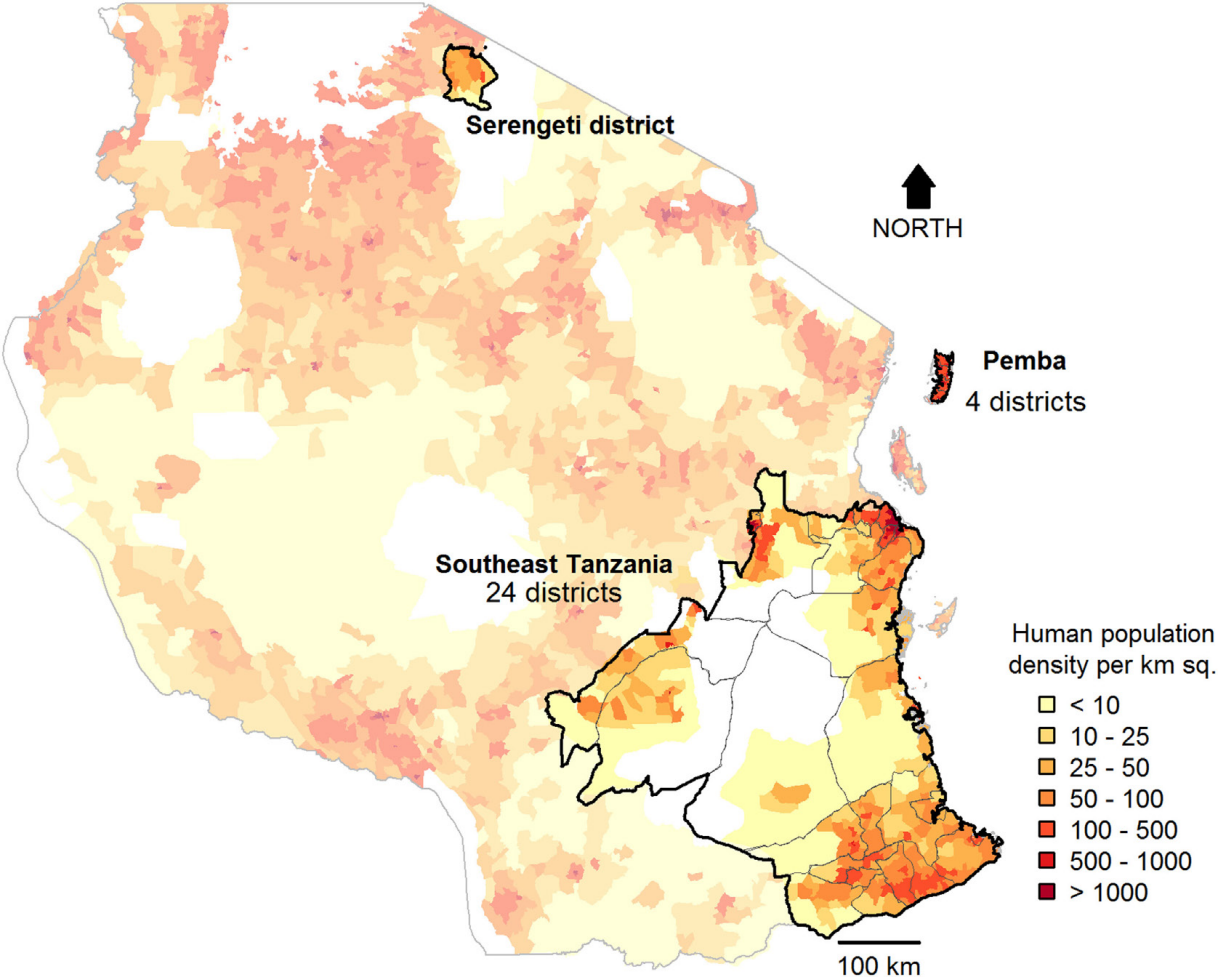
**Study sites in Tanzania**. Post-vaccination transects (2 sub-villages/village in 2,070 villages), school-based surveys (6 schools/district), and household surveys (30 households/village in 6 villages/district) were conducted in southeast Tanzania and Pemba. In Serengeti district, transects were conducted in all sub-villages in almost all villages (85/88), and four school-based surveys and a complete census of dogs (surveys of 35,867 households) were undertaken. Km sq, Square Kilometres.

The study was conducted in 29 districts across Tanzania: 24 districts from southeast Tanzania, 4 districts from Pemba island, and 1 district (Serengeti district) from northwest Tanzania (Figure 1). These areas are inhabited by an estimated 9.1 million people (20% of the Tanzanian population) according to the 2012 national census (20) and represent districts that span a wide range of settings, comprising rural, urban, coastal and inland areas, and a range of livelihoods and religious backgrounds. Mass dog vaccination campaigns were conducted in all these districts by local government teams, with support of WHO and collaborating institutions. Various methods of estimating vaccination coverages achieved during campaigns were compared. Table 1 summarizes the methods used in different locations and the rationale for data collection.

**Table 1:** **Study design and data collection including purpose of each dataset**.

Method	Areas (number of villages)	Sampling design	Data collection period	Interval between village-level campaign and coverage survey	Purpose
Post-vaccination transects	Serengeti (85)	1 transect in every sub-village (357 total) in all villages	May–October 2015	2–3 h	Coverage estimates at village and district level. Data used for simulations to explore how the number of transects/village affect precision of district-level estimates

	Southeast Tanzania and Pemba (2,070)	1 transect in 2 sub-villages (4,140 total) in every village/district	November 2014–January 2015	2–3 h	Setup and implementation costs

School-based surveys	Serengeti (4)	100 pupils/school in 4 schools/district (333 pupils)	July 2015	1 month	Coverage estimates at district level. Precision of estimates compared with census data and simulation experiments.

	Southeast Tanzania and Pemba (115)	100 pupils/school in 6 schools/district (8,254 pupils)	November 2014 and February 2015	1–2 months	Setup and implementation costs

Household survey	Southeast Tanzania and Pemba (160)	30 households/village in 6 villages/district (4,488 households)	July–August 2011	2–6 months	Setup and implementation costs. Data used to parameterize simulations for settings with high: human dog ratios to explore precision of household surveys

Complete human and dog census	Serengeti (88)	All households in district (35,867)	From 2008 to 2015	Vaccination campaigns ~May–July each year. Census at different times of year for each village	Census does not provide a point estimate of coverage relative to a specific campaign. Data used for simulation experiment to determine how sampling (e.g., household and school-based surveys) affects precision of coverage estimates

### Post-Vaccination Transects

To generate rapid estimates of village-level vaccination coverage, post-vaccination transects were conducted on the same day as vaccination campaigns in each village from 4 p.m. to 6 p.m. when dogs were active and visible. Transects involved recording all dogs observed while walking (or occasionally cycling) a route through villages counting marked (vaccinated) and unmarked (unvaccinated) dogs. In rural communities, transects were conducted in two randomly selected sub-villages from each village (villages ranged in size from 2 to 10 sub-villages, with a median of 4 sub-villages/village), aiming to representatively sample coverage within each village. In the first sub-village, enumerators were instructed to start transects at the center of the sub-village heading to the outskirts, while in the other sub-village, transects started from the edge of the sub-village and headed toward the center. Each transect was conducted by one enumerator for 1 h, therefore, taking a total of 2 h to complete each village. In urban areas, enumerators were required to cover the jurisdiction of a street (a geographical area defined from the National Census, which covers a neighborhood with several roads). One day of training was held for enumerators prior to data collection and printed protocols, and data collection.

Printed protocols and data collection forms were provided to enumerators during this training. Enumerators selected the direction at the start of transects, at the border of sub-villages/streets and at road junctions by spinning a pen. In Serengeti district, transects were conducted in every sub-village of vaccinated villages.

### School-Based Surveys

School-based surveys were conducted within 2 months of vaccination campaigns in southeast Tanzania, Pemba, and Serengeti district (Table 1). In each district in southeast Tanzania and Pemba, six primary schools (one school per village, as most villages in Tanzania have a primary school) were randomly selected, and in Serengeti district, four primary schools were selected. Logistic and financial limitations meant that school surveys were not conducted in some districts or were conducted in less than six schools per district as initially planned. Between 50 and 100 pupils (one per household) from Standard IV–VII (aged 11–15 years) were asked to complete a questionnaire to collect data from their household. We used total population purposive sampling with a target to interview 100 pupils per school. This resulted in all Standard VII pupils being selected to fill the questionnaire. If there was more than one pupil from one household recruited, the oldest was selected. If the school had fewer than 100 standard VII pupils, pupils were recruited from lower classes (Standard IV–VI). Written consent from the district executive officer and verbal consent of teachers and pupils were obtained at each primary school prior to the study. To introduce the project to schools, researchers were accompanied by the district veterinary officer, district health officer, and district education officer. Questionnaires were administered to pupils by the lead author and his research team. The questionnaire included questions on the number of adults and children (<18 years of age) living in the household, the number of dogs and puppies (<3 months of age) kept at the household, and the age of dogs and their vaccination status.

### Household Surveys

Household surveys were conducted in all districts in southeast Tanzania and Pemba with the aim of obtaining an initial assessment of coverage from the first phase of vaccination campaigns. Six villages were randomly selected from all villages in each district, and the survey was conducted by surveying 30 households in each of the selected villages. In every randomly selected village, a landmark was identified (preferably a school, otherwise a dispensary, church, or mosque). From this starting point, interviewers randomly chose a direction for selecting households for interview by spinning a pen. Every third household was sampled, and interviews conducted until 30 households were completed in each village. Surveys were conducted in July and August 2011, around 4 months after dog vaccination campaigns conducted in March and April 2011. Interviewers were accompanied by local village officers to identify household heads and provide introductions. Prior to the administration of the questionnaire, permission was sought from the household head or other household members of at least 18 years of age in the absence of the household head. Interviewers explained the study background to each respondent and obtained verbal consent to carry out the questionnaire. For households that owned dogs, the questionnaire captured details of dogs owned (adults and puppies <3 months) and their vaccination status on the basis of owner recall.

### Serengeti District Dog Population Census

In Serengeti district, a complete census was conducted to collect the same household questionnaire data as described above, for every household in the district. The census began in 2008 and was completed in 2015 (Table 1), as enumerators were only able to conduct the census in between other activities. Because the census was conducted over an extended period, it was not used to generate point estimates of vaccination coverage in relation to specific vaccination campaigns, which in Serengeti have been conducted annually over the last decade. Instead these data were used for a simulation experiment, whereby the data were sampled to simulate a household survey, thereby enabling a comparison of methods and how they affect the precision of coverage estimates (see Data Analysis).

### Resources for Estimating Vaccination Coverage

The number of people involved in each survey method, the time required to complete data collection and associated costs to set up and implement each assessment across southeast Tanzania were recorded. Costs per surveyed village were calculated as total costs incurred in all districts divided by the number of villages surveyed. Costs per district were calculated as the overall costs for conducting the surveys across surveyed districts, divided by the number of surveyed districts. The costs incurred included per diems to government officials such as District Veterinary Officers, District Health Officers, District Education Officers, and researchers and allowances to enumerators who conducted transects. Communication costs covered phone calls to coordinate with enumerators and data collectors. Fares covered travel to districts to facilitate training, supervision, and to collect records. For school-based and household surveys, travel covered fuel for vehicle use. All costs were calculated for evaluation of a single mass dog vaccination campaign in Tanzanian shillings (TZS) and converted to US dollars (US$) using the average exchange rate in 2011 [1 TZS to US$ 0.000632 (21)].

### Data Analysis

The census data from Serengeti district together with the transects and school-based surveys conducted in Serengeti in 2015 were used to determine the impacts of sampling on the precision of vaccination coverage estimation. We define accuracy as lack of bias. Repeated estimates using an accurate method will converge on the true coverage value as sample size increases. Precision is the absence of random sampling error from the measured value. Repeated estimates using a precise method will be close to their mean, although not necessarily close to the true coverage. Clearly, for an estimation method to be informative about the true coverage, it must be both accurate and precise. Across Tanzania there is considerable variation in dog ownership, from largely Muslim communities with very few dogs per household to pastoralists with many dogs in most households. This variation in dog ownership patterns among communities means that sampling designs should aim to deal with these variations and give accurate and precise estimates.

**Figure 2:**
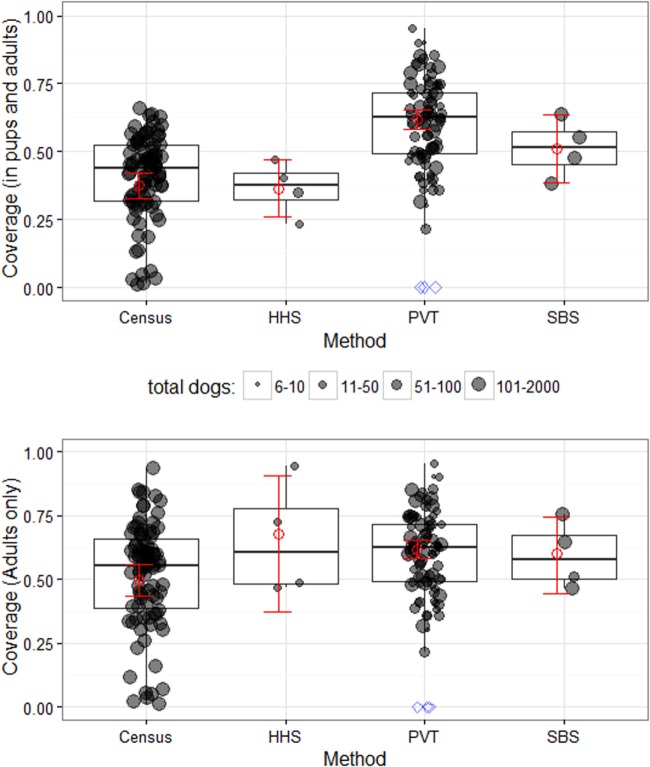
**District and village-level vaccination coverage estimates and precision in Serengeti District**. Coverage estimates are shown for all dogs (including puppies, top) and adult dogs only (bottom) in surveyed villages (dots); the dots also represent the village-level coverage. Red squares and error bars show mean district-level coverage ±95% CI, estimated using generalized linear mixed models (see main text for details). The coverage distribution is plotted for individual villages (shaded circles) and summarized by box-and-whisker plots, where the thick line shows the median, the box covers the interquartile range and the whiskers extend to the range. Blue diamonds represent villages with no vaccination campaign where vaccination coverage was assumed to be zero (not included in calculation of mean ± 95% CI or boxplots). PVT, post-vaccination transects; SBS, school-based surveys; HHS, household surveys.

To examine the precision of different methods in estimating vaccination coverage, we estimated the district-wide mean coverage and 95% confidence intervals in Serengeti from the complete census (all households in all 88 villages) and from subsamples of households and villages from the census equivalent to a household survey. We also compared these to the precision of district-wide coverage estimates from the school-based surveys (in 4 villages) and post-vaccination transects (in 85 villages) in Serengeti district. To facilitate comparison, the four villages selected for the household survey during the simulation in Figure 2 were the same ones sampled by the school-based survey. We fitted binomial generalized linear mixed models (GLMMs), with a random intercept to account for variation in mean coverage between villages.

To assess the impact of sampling on district-wide coverage estimates, we conducted simulations where we subsampled from the complete census (88 villages) different numbers of households per village (10, 20, 30, 40, and 50) and villages (5, 10, 20, 30, 40, 50, 60, 70, 80, and 88). Each of the 50 combinations of these two sampling choices was simulated 500 times, and mean coverage for the district was estimated from each simulated data set as the total number of vaccinated dogs divided by the total number of dogs. Although generally this simple method is inferior to fitting a GLMM as above (22), this was not feasible for sampling designs with low total dog numbers. The precision achieved using each sampling design was assessed by plotting coverage estimates against the numbers of villages and households sampled.

To assess the impact of variability in dog ownership or HDR on the precision of coverage estimates, we repeated the simulation described above. However, instead of subsampling from the Serengeti census dataset, we used a simulated dataset with the same structure but with fewer dogs per household. The number of dogs in each household was simulated from a negative binomial distribution with mean μ = 0.2 and dispersion parameter *k* = 0.06 [calculated from the mean and variance of the household survey data in southeast Tanzania using the parameterization of the negative binomial with variance μ + (μ^2^/k)]. The number of vaccinated dogs was simulated with mean coverage and random effect variances between villages, sub-villages, and households estimated from a binomial GLMM fitted to the Serengeti census dataset. As a result, the “low dog ownership” dataset was as similar as possible to (and therefore comparable to) the Serengeti dataset, but with dog numbers similar to the mean dogs/household in southeast Tanzania (Table 1). As the results presented here come from a single simulated “low dog ownership” dataset, we checked for sensitivity to random variation by comparing across several (>5) simulated data sets. We also assessed the impact of sampling using transect surveys. We examined the scenario of sampling 1, 2, 4 and 8 (or all if <8) sub-villages in a village and determined which sampling effort (sampling design) provided reasonable estimates of village-level coverage.

All statistical analyses were conducted using R version 3.3.1 (23). GLMMs were fitted using the *lme4* package (24), and the “low dog ownership” data set was simulated using the *sim.glmm* function (25).

### Ethical Considerations

We obtained ethics approval from the Medical Research Coordinating Committee of the National Institute for Medical Research of Tanzania (NIMR/HQ/R.8a/Vol.IX/2109) and Tanzania Commission for Science and Technology (COSTECH). Before administering any questionnaires, participants were informed about the background and purpose of the study, highlighting that their participation was voluntary, and that their answers would be kept confidential. Only participants who verbally agreed were interviewed.

## Results

Across southeast Tanzania, Pemba Island and Serengeti district, we conducted (i) post-vaccination transects following vaccination campaigns in 2,155 villages and counted 24,721 dogs, (ii) questionnaires with 8,587 primary school pupil respondents, each representing a unique household, in 119 randomly selected schools (3,090 dogs recorded), and (iii) 4,488 household surveys in 160 randomly selected villages (731 dogs recorded—excluding Serengeti district). In addition, a complete census was conducted in Serengeti district covering 35,867 households, which collectively owned 62,771 dogs (Table 1). Table 2 summarizes the attributes of each study district and dogs recorded by each method. Many more dogs were observed on transects than were recorded in either household or school-based surveys, even in districts with low dog ownership i.e., high HDR (Table 2).

**Table 2:** **Descriptive characteristics of the study districts**.

			Post-vaccination transects			Household survey				School-based survey			
District	Setting (Urban/Rural, Coastal/inland, island)	Total villages (or wards)	Villages/streets surveyed	Dogs sighted (Village mean)	Villages with no dogs seen	Villages surveyed	Households (HH) surveyed	Dogs recorded (mean dogs/HH)	Households without dogs	Schools surveyed	Pupil respondants	Dogs recorded (mean dogs/family)	Families without dogs
Chake Chake	Island	29	29	182 (6.28)	0	6	178	7 (0.04)	176	3	152	3 (0.02)	151
Ilala	Urban coastal	26	NA	NA	NA	6	133	34 (0.26.)	119	NA	NA	NA	NA
Kibaha Rural	Rural inland	55	55	759 (13.80)	0	2	93	30 (0.32)	82	6	412	199 (0.50)	355
Kibaha Urban	Urban inland	50	50	526 (7.21)	0	6	151	66 (0.44)	117	6	407	237 (0.51)	341
Kilombero	Rural inland	80	77	1,989 (25.83)	0	6	147	32 (0.22)	132	6	548	218 (0.40)	470
Kilwa	Rural coastal	102	78	606 (7.77)	4	6	158	26 (0.16)	144	NA	NA	NA	NA
Kinondoni	Urban coastal	34	83	349 (4.20)	19	6	183	59 (0.32)	154	6	471	163 (0.35)	430
Kisarawe	Rural inland	77	77	578 (7.41)	1	6	170	9 (0.05)	163	6	283	109 (0.39)	230
Lindi Rural	Rural coastal	134	134	1,754 (10.83)	8	6	177	15 (0.08)	168	5	254	60 (0.24)	242
Lindi Urban	Urban coastal	30	60	588 (9.80)	2	6	177	17 (0.10)	168	4	343	70 (0.20)	316
Liwale	Rural inland	76	73	531 (7.27)	6	6	175	19 (0.11)	169	NA	NA	NA	NA
Masasi	Rural inland	159	97	554 (6.16)	5	6	180	27 (0.15)	162	3	161	32 (0.20)	147
Micheweni	Island	27	27	178 (6.59)	0	6	173	25 (0.14)	164	3	156	4 (0.03)	155
Mkoani	Island	33	33	303 (9.18)	4	6	154	9 (0.06)	151	3	177	8 (0.05)	175
Mkuranga	Rural coastal	116	90	262 (2.91)	30	6	174	4 (0.02)	171	6	328	58 (0.18)	306
Morogoro Rural	Rural inland	144	93	1,056 (12.00)	15	6	168	41 (0.24)	145	5	393	103 (0.25)	356
Morogoro Urban	Urban inland	19	163	572 (3.51)	1	6	169	49 (0.29)	146	6	557	225 (0.40)	489
Mtwara Rural	Rural inland	156	85	427 (5.02)	16	5	140	16 (0.11)	138	5	334	31 (0.09)	328
Mtwara Urban	Urban coastal	86	15	148 (9.87)	1	6	150	14 (0.09)	130	3	288	69 (0.24)	266
Nachingwea	Rural inland	118	115	1,576 (13.70)	4	6	170	37 (0.22)	160	6	342	84 (0.25)	307
Nanyumbu	Rural inland	89	58	415 (7.16)	10	6	176	1 (0.01)	175	6	475	28 (0.06)	466
Newala	Rural inland	153	83	626 (7.54)	1	6	180	4 (0.02)	178	6	645	55 (0.09)	623
Ruangwa	Rural inland	89	79	758 (9.59)	1	6	179	37 (0.21)	164	4	168	24 (0.14)	156
Rufiji	Rural coastal	115	78	470 (6.03)	16	6	172	2 (0.01)	171	5	459	61 (0.14)	427
Tandahimba	Rural inland	156	130	360 (2.77)	42	3	79	2 (0.03)	78	3	175	24 (0.14)	170
Temeke	Urban coastal	30	106	276 (2.60)	19	6	159	8 (0.05)	155	NA	NA	NA	NA
Ulanga	Rural inland	70	70	2,381 (28.35)	0	6	177	85 (0.48)	146	6	560	326 (0.58)	464
Wete	Island	32	32	213 (6.63)	2	6	146	56 (0.38)	124	3	166	7 (0.04)	162
Serengeti	Rural	88	85	6,285 (35.21)	0	4^a^	120^a^	179 (0.37)^a^	0^a^	4	333	892 (2.68)	51

*In urban areas the numbers of wards are listed per district rather than villages*.

*^a^In Serengeti district a simulated household survey dataset was generated from a subsample of 120 households from four villages (30 households per village) of the complete census data*.

*NA, not available*.

### Logistics and Costs for Coverage Assessments

Post-vaccination transects usually took around 2 h to complete. Collars were fitted to dogs during vaccination campaigns with very few cases where this was not possible. As transects were conducted the same day as campaigns, collar loss was assumed to be negligible. School-based surveys involved two research scientists with the help of teachers. The questionnaire was administered in one classroom, and all pupils normally took approximately 40 min to complete questionnaires. Household surveys involved a research team comprised of two drivers, eight interviewers, and one supervisor split between two vehicles. Each vehicle covered four villages per day (an average of one village per interviewer/day), and the village leader accompanied each interviewer in every village. The census in Serengeti district was the most time-consuming method, with locally trained interviewers spending an average of 14 (8 h/day) days to complete a census of one village.

Costs of estimating coverage varied depending upon the method. The costs per village were $12.01, $66.12, and $155.70 for transects, school-based, and household surveys, respectively, and these costs scaled up with the sampling for each method (Table 3). Specifically, the average cost for assessing district-level coverage was around $1,300 with transects completed in every village, approximately $300 based on 6 school-based surveys per district and $900 based on sampling 30 households per village in six villages per district.

**Table 3:** **Cost comparison between methods of evaluating dog vaccination campaigns in Southeast Tanzania and Pemba island**.

	Cost item	Transects (*n* = 2,070)		School-based surveys (*n* = 115)		Household surveys (*n* = 160)	
Setup		Total cost ($)	Cost/village ($)	Total cost ($)	Cost/village ($)	Total cost ($)	Cost/village ($)
	Communication costs	606.08	0.29	20.01	0.17		
	Fare	613.02	0.3				
	Training/supervision	2,256.28	1.09	4,203.06	36.55		
**Subtotal (setup costs)**			**$1.68**		**$36.72**		
Implementation	Per diems/allowances	6,541.2	3.16	624.45	5.43	21,345.30	133.41
	Data collection	176.80	0.09			659.5	4.12
	Collars	13,858.09	6.69				
	Questionnaire	806.16	0.39	1,200.88	10.44		
	Fuel			1,555.64	13.53	2,992.92	18.17
**Subtotal (implementation costs)**		**$10.33**		**$29.40**		**$155.70**
**Cost per village**			**$12.01**		**$66.12**		**$155.70**
**Cost per district**		$1,307.37		$310.60		$889.05	

*The numbers of villages and districts which these calculations were based on are shown in Table 1. All costs are in USD. Per diems for household surveys covered supervisors, drivers, village leaders, and researchers. Allowances for enumerators conducting transects were $3.16/village. Household survey costs were based on interviewing 30 households per village. Data collection for household surveys also included the cost of mobile phones used by researchers for submitting data (six phones at $94.8/phone)*.

### Comparison of Coverage Estimates and Their Precision between Methods

Vaccination coverage in Serengeti district was estimated using each method and from the complete census to assess precision in coverage estimates. Figure 2 illustrates village-level coverage estimates and the district-wide mean estimates. Transects in Serengeti were conducted in 85 out of 88 villages, with 6,285 dogs counted and school-based surveys were conducted in four schools, with interviewed pupils representing 333 households and collective ownership of 892 dogs. We observed that excluding puppies resulted in higher estimates of coverage (from 37.5% as estimated from the census including puppies and adults to 49.7% including only dogs >3 months of age), with similar increases for both the household and school-based surveys. However, we were unable to analyse the post-vaccination transect data according to age class of observed dogs as this information was not recorded during transects.

Our GLMM estimate of district-level coverage of all dogs (puppies and adults) from the census was 37.5% with relatively narrow 95% confidence intervals (32.8–42.3%). The coverage estimate from the census data subsampled to represent a household survey fell outside of these confidence intervals at 44.5% and had wider 95% CI (37.1–52.0%). Although the district-wide coverage for the school-based survey (51.2%) was not directly comparable to the census data, the span of the 95%CI can be compared and was found to be much wider (38.7–63.4%). The transect coverage estimate (61.7%) was higher than the school-based survey but had narrow 95% CI (58.2–65.2%) similar in span to the census.

In comparison to the census, only the post-vaccination transects method provided similar precision in coverage estimates (Figure 2) but these appeared to overestimate district-level vaccination coverage in comparison to the school-based survey. This is likely due to few puppies being observed during the transects. Transects generated coverage estimates for every village in a district, although village-level estimates were not very precise. Nonetheless, these village-level estimates were sufficient for identifying villages with low coverage, for example, less than 70% coverage.

### Impact of Sampling on District-Level Coverage Estimates

**Figure 3:**
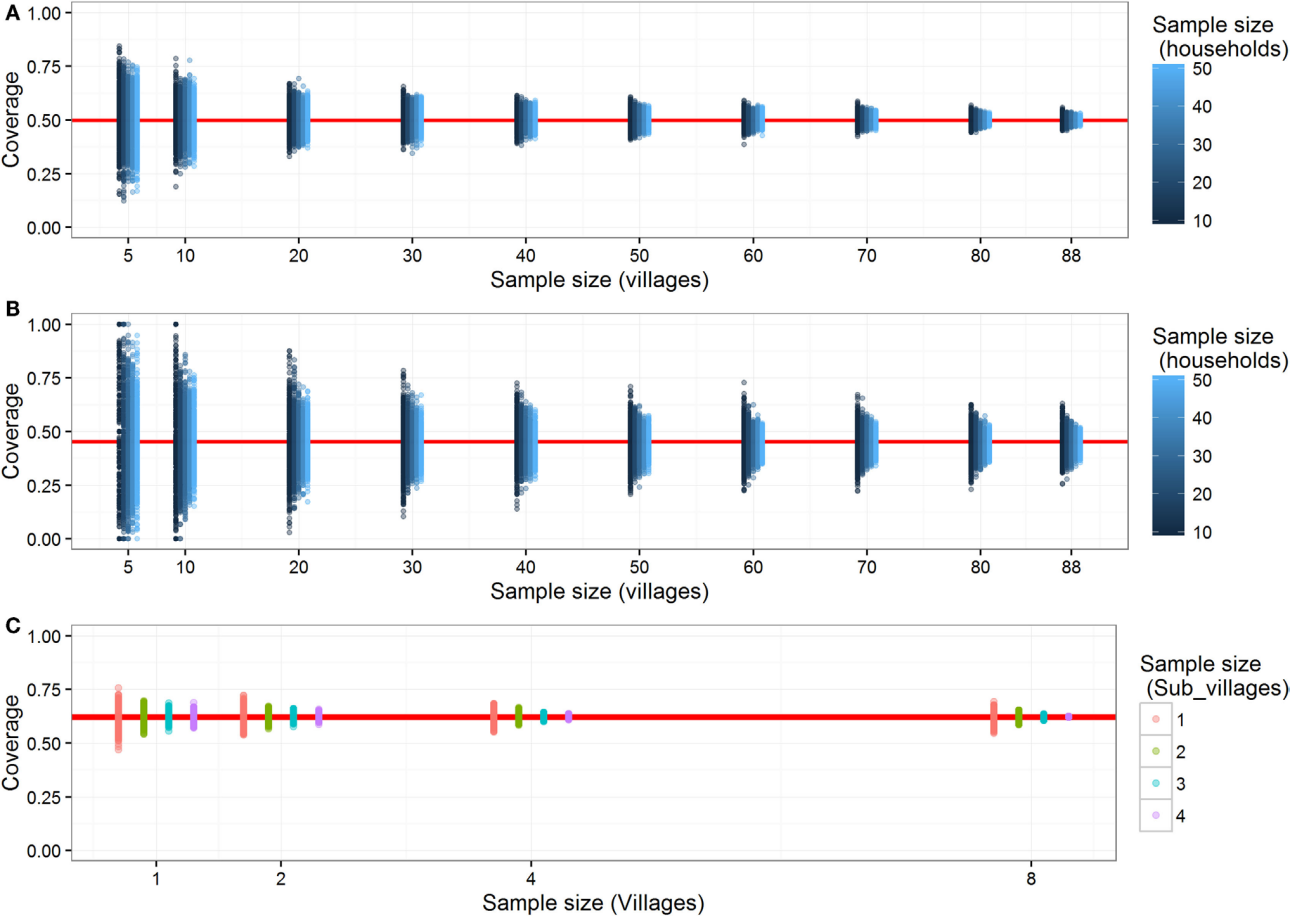
**The impact of sampling on precision of coverage estimates derived from household surveys in communities with (A) low human:dog ratios and, (B) high human:dog ratios, and from (C) post-vaccination transects**. Estimated mean district-level vaccination coverage (red line) for different numbers of villages and households sampled from **(A)** actual Serengeti district dataset and **(B)** a dataset from Serengeti District but simulated with lower dog ownership (0.2 dogs per household). For each sampling design [i.e., the number of villages and households sampled in panels **(A,B)**], coverage estimates from 500 subsampled data sets are plotted (blue dots), with shading indicating the number of sampled households, and the mean of these estimates is shown by red line. Similar to panels **(A,B)**, each column of points shows sampling variation among 500 subsampled data sets for each sampling design using transects **(C)**. Coloured dots represent the number of subvillages sampled per village for estimating coverage from transects.

Estimates of coverage from the school-based and household surveys were sensitive to the sampling design (Figure 3). As the sample size increases, in terms of the numbers of households sampled per village, coverage estimates became increasingly precise (Figure 3A). In Serengeti district, where there is high dog ownership, once at least 30 households within each of 20 villages were sampled, estimates were very close (±10% with high probability) to the true mean from the census data. In scenarios with low dog ownership (i.e., higher HDR), approximately three times the sampling effort (30 households × 60 villages) is required to achieve an equivalent degree of precision (Figure 3B). It was possible to sample more households more rapidly through school-based surveys than household surveys because it is easier to recruit pupils at school than visiting individual households.

For the transects, sampling two or more sub-villages per village gave coverage estimates that were within 10% of the true village-level coverage, although coverage estimates were more precise if transects were completed in all villages in all wards rather than just a sample of villages per ward (Figure 3C).

## Discussion

The feasibility of global canine rabies elimination has been recognized by major international health agencies, including the WHO, the World Animal Health Organization (OIE), and the Food and Agriculture Organization of the United Nations (FAO) (5). Implementation of mass dog vaccination programs to meet the 2030 target of zero human deaths are now underway in several countries in Asia and Africa. To guide the progress of these programs, it is important to evaluate the performance of mass dog vaccination campaigns. Specifically, monitoring is useful to determine whether campaigns have reached the required vaccination coverage, to identify problematic areas with low coverage, and target communities that have been missed with intensified vaccination effort. Dog rabies control programs typically operate under financial constraints that affect both implementation and evaluation. While several studies have evaluated vaccination coverage as part of small-scale research/pilot vaccination campaigns (26), here we evaluate different approaches in the context of comparison of setup and implementation costs for generating precise and accurate coverage estimates at scale.

In this study, we demonstrated that transects were the simplest method that generated precise estimates of vaccination coverage and were also not cost prohibitive. A limitation of transects is that they tend to overestimate coverage. It was previously reported that post-vaccination coverage estimates in Tanzania from transects overestimate coverage by 10–15% (19). We saw a similar difference in our coverage estimates from the complete census when puppies were excluded. This suggests that puppies are rarely observed on transects and that puppies are less likely to be vaccinated, which could explain why coverage is overestimated from transects (19). Estimates of vaccination coverage from transects should therefore be reduced by around 10% when assessing whether coverage is sufficient or if remedial vaccination is required, and for determining the impacts of vaccination programs.

Household surveys generate useful data on vaccination coverage of owned dogs and provide opportunities for collection of additional demographic data (15, 18, 19). However, we found that household surveys were time consuming and costly at ~$150 per village. Because of these costs, we restricted out household (and school-based) surveys to a set number (6) per district, which meant that larger districts were sampled less. However, we found that approximately 30 villages would need to be surveyed to generate district-level estimates of coverage precise to within 10% of the true coverage. We conclude from our simulations that the sampling required to reach an adequate level of precision (say within 5%) would likely be cost prohibitive in most settings, particularly where HDRs are high and even larger sample sizes would be needed. The effort required to conduct these surveys would be difficult to justify, given the more urgent priority of vaccinating dogs.

School-based surveys can generate data from more households at lower cost, as pupils are easily recruited. Moreover, school pupils typically take their dogs to vaccination stations and, therefore, know the vaccination status of their dogs (18). The main costs of school-based surveys are at the setup stage, which requires considerable government support, although this cost is not incurred on successive campaigns. School-based surveys are, therefore, simple to implement and can capture a range of socioeconomic and religious backgrounds. However, estimates may be less accurate because of a biased subsample of children attend school and less precise in areas with low numbers of pupils attending schools, such as pastoralist communities. Critically, this method may, therefore, fail to capture coverage in the most vulnerable populations with the highest dog ownership (lowest HDR) but lowest school attendance (27). In communities with few dogs, school-based surveys are also sensitive to sampling, as very few pupils (<10 pupils per 100 households) reported to own dogs at their households (see also simulation experiments in Figure 3B). In these areas, large numbers of schools would need to be surveyed to obtain sufficient sample sizes for adequately precise coverage estimates.

**Figure 4:**
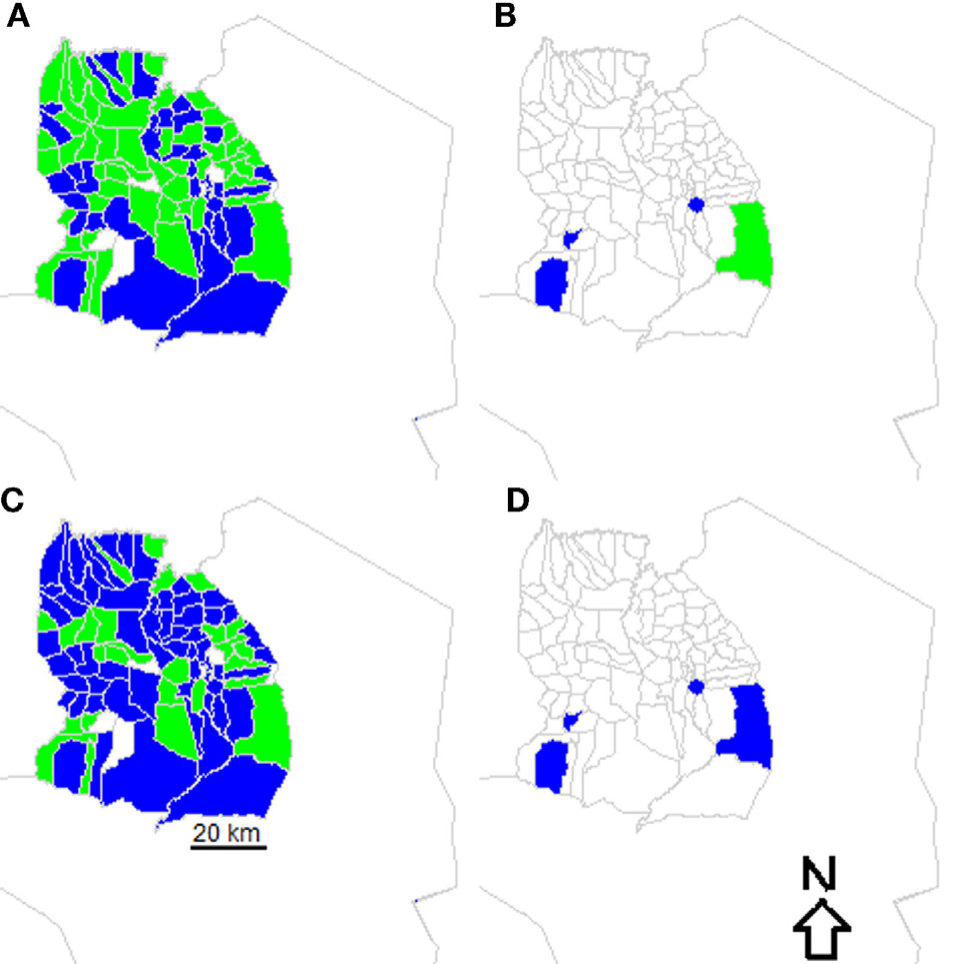
**Vaccination performance in villages in Serengeti District**. Villages where surveys were conducted are colored based on whether village-level coverage exceeded 60% (green) or were less than 60% (blue) based on **(A)** post-vaccination transects and **(B)** school-based surveys versus whether coverage exceeded 70% (green) or were less than 70% (blue) based on **(C)** post-vaccination transects and **(D)** school-based surveys.

Among the limitations of our household and school-based surveys was their timeliness; we also used the vaccination status of dogs reported by owners, which could be biased. More logistic effort was involved in setting up these surveys than for transects, therefore rapid assessments of vaccination performance (and remedial action if required) are more difficult with these methods, which also do not provide estimates of coverage for every village unless completed in every village which would be very costly. By contrast, transects were very efficient and generated immediate operational guidance at the village-level (Figure 4).

On the whole, many more dogs were recorded by transects than other methods. For example, fewer than 10 dogs were counted during household surveys in Chake Chake district on Pemba, while 182 dogs were counted during transects. Transects surveys are therefore more likely to generate more precise estimates of coverage than the other methods even in areas with fewer dogs. However, at the village-level dog counts even from transects were often very low and therefore village-level coverage estimates would be expected to be imprecise. Although transects could be carried out for longer periods of time, this might also result in recounting of dogs, and would make them more expensive to conduct. Overall, transects were affordable and generated more precise estimates of district-level coverage than questionnaire-based surveys that were affected by sampling. But costs of transects accrue as more villages are surveyed, so in very large populations (with lots of villages) the costs of transects increase.

Priorities in terms of vaccination campaign evaluation typically change over time (28). During initial stages of national control programs, the priority, for example, is likely to be planning for dog vaccine procurement, with estimates needed of the dog population size. Human census data are almost universally available and can be used with HDRs to provide a baseline for vaccine procurement (29). HDRs for a range of settings in Africa and Asia are a useful starting point (7, 15, 26). However, these data should not be considered sufficiently reliable to provide a denominator for generating vaccination coverage estimates. Indeed, our experience in southeast Tanzania was that dog population estimates derived from HDRs substantially overestimated dog populations and reassessment of vaccine procurement was required in subsequent years. But, in general, it was better to overestimate the dog population at this stage than underestimate it.

Consecutive vaccination campaigns should generate data on vaccine doses delivered at the village level. We therefore recommend post-vaccination transects be used in conjunction with monitoring vaccine doses delivered during campaigns to guide vaccine procurement for future campaigns. This approach may mean that once baseline levels of coverage have been established through accurate records of dogs vaccinated in each village/vaccination station, post-vaccination transects may not be required every year, but could be completed less frequently. In our experience, local government authorities in Tanzania do not have resources or incentives to invest in monitoring and evaluation, and their priority, understandably, is on vaccinating dogs. A further advantage of post-vaccination transects is that local paravets, community-based health officers, local community members, and volunteers can be rapidly trained to conduct transects and therefore provide relatively independent coverage data.

A major obstacle when approaching elimination is the need to address difficulties in program implementation in hard-to-reach populations (30). Post-vaccination transects could be used to troubleshoot the performance of vaccination coverage in stubborn foci. For example, vaccination programs across Latin America have achieved tremendous success in controlling dog rabies with average levels of coverage estimated to exceed 70% based on HDRs (31). However, in localized areas canine rabies persists, likely due to gaps in coverage or overestimation of routine coverage achieved (32). Transects could be used to identify areas in need of improved vaccination, where delivery was poor (for example in Figure 4). More generally, transects have proven to be effective in measuring the immediate success of vaccination campaigns in settings in both Asia and Africa (12–14, 29, 33). One concern is that transect routes are not pre-defined, which could result in recounting of dogs. But efforts can be taken to avoid recounting dogs, as we did by aiming to go from the outskirts to the center of sub-villages and vice versa. In our study, some enumerators cycled rather than walked transects, but enumerators were trained to cover routes slowly for 1 h, so we expect that any differences due to this would have been negligible. Simple tools are available to evaluate the performance of vaccination programs, capturing the spatial variation that transects provide, which could also address these concerns (29).

Patterns of dog ownership in Tanzania are very heterogeneous. As such, district-level coverage estimates from household or school-based surveys tend to be more imprecise than estimates from transects. To obtain estimates with comparable precision would require considerable increased sampling and costs. Moreover, from transects we were able to estimate village-level coverages. This can be useful when aiming to eliminate rabies as gaps in coverage can be detected, and therefore campaigns can be strengthened to effectively interrupt transmission. With the wide availability of mobile phones, real-time data on vaccinated dogs and coverage estimates from transects can easily be submitted by enumerators (29, 34). We therefore recommend transects as a relatively cheap method to estimate village-level coverage that can be conducted at scale, in comparison to other methods where high levels of sampling are required that are cost prohibitive.

## Author Contributions

Conceived and designed experiments: MS, JC, SC, TL, KL, ZM, MM, LS, and KH; performed experiments: MS, JC, AL, KL, MM, EM, ZM, LS, and KH; developed analytical tools: MS, PJ, and KH; and wrote the paper: MS, PJ, KHo, JC, TL, AL, KL, MM, EM, ZM, LS, and KH.

## Conflict of Interest Statement

The authors declare that the research was conducted in the absence of any commercial or financial relationships that could be construed as a potential conflict of interest.
